# Dimanganese(II) hydroxide vanadate, Mn_2_(OH)[VO_4_]

**DOI:** 10.1107/S1600536814012926

**Published:** 2014-06-11

**Authors:** Kewen Sun, Angela Möller

**Affiliations:** a 112 Fleming Building, Department of Chemistry, University of Houston, Houston, TX 77204-5003, USA

## Abstract

Dimanganese(II) hydroxide vanadate was obtained from hydro­thermal reactions. The crystal structure of the title compound is isotypic with that of Zn_2_(OH)[VO_4_]. Three crystallographically independent Mn^2+^ ions are present, one (site symmetry .*m*.) with a distorted trigonal-bipyramidal and two (site symmetries .*m*. and 1) with distorted octa­hedral coordination spheres. These polyhedra are linked through common edges, forming a corrugated layer-type of structure extending parallel to (100). A three-dimensional framework results *via* additional Mn—O—V—O—Mn connectivities involving the two different tetra­hedral [VO_4_] units (each with point-group symmetry .*m*.). O—H⋯O hydrogen bonds (one bifurcated) between the OH functions (both with point-group symmetry .*m*.) and the [VO_4_] units complete this arrangement.

## Related literature   

Mn_2_(OH)[VO_4_] is isotypic with Zn_2_(OH)[VO_4_] (Wang *et al.*, 1998 ▶), Zn_1.86_Cd_0.14_(OH)[VO_4_] (Đorđević *et al.*, 2010 ▶), Cu_2_(OH)[VO_4_] (Wu *et al.*, 2003 ▶), but not with the acentric Cu polymorph reported by Zhang *et al.* (2014 ▶).

## Experimental   

### 

#### Crystal data  

Mn_2_(OH)[VO_4_]
*M* *_r_* = 241.83
Orthorhombic, 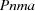

*a* = 14.9112 (10) Å
*b* = 6.1225 (3) Å
*c* = 9.1635 (4) Å
*V* = 836.57 (8) Å^3^
*Z* = 8
Mo *K*α radiation
μ = 8.04 mm^−1^
*T* = 293 K
0.14 × 0.02 × 0.02 mm

#### Data collection  

Rigaku R-AXIS conversion diffractometer
Absorption correction: multi-scan (*CrystalClear*; Rigaku, 2011 ▶) *T*_min_ = 0.777, *T*_max_ = 1.000
11657 measured reflections
1715 independent reflections
1637 reflections with *I* > 2σ(*I*)
*R*_int_ = 0.046

#### Refinement  

*R*[*F* ^2^ > 2σ(*F* ^2^)] = 0.045
*wR*(*F* ^2^) = 0.062
*S* = 1.36
1715 reflections
92 parameters
2 restraints
H atoms treated by a mixture of independent and constrained refinement
Δρ_max_ = 0.74 e Å^−3^
Δρ_min_ = −0.84 e Å^−3^

### 

Data collection: *CrystalClear* (Rigaku, 2011 ▶); cell refinement: *CrystalClear*; data reduction: *CrystalClear*; program(s) used to solve structure: *SHELXS97* (Sheldrick, 2008 ▶); program(s) used to refine structure: *SHELXL97* (Sheldrick, 2008 ▶); molecular graphics: *DIAMOND* (Brandenburg, 2012 ▶); software used to prepare material for publication: *publCIF* (Westrip, 2010 ▶).

## Supplementary Material

Crystal structure: contains datablock(s) I, New_Global_Publ_Block. DOI: 10.1107/S1600536814012926/wm5026sup1.cif

Structure factors: contains datablock(s) I. DOI: 10.1107/S1600536814012926/wm5026Isup2.hkl

CCDC reference: 1006602

Additional supporting information:  crystallographic information; 3D view; checkCIF report

## Figures and Tables

**Table 1:** Hydrogen-bond geometry (Å, °)

*D*—H⋯*A*	*D*—H	H⋯*A*	*D*⋯*A*	*D*—H⋯*A*
O3—H3⋯O12	0.96 (2)	1.79 (2)	2.734 (4)	165 (5)
O4—H4⋯O11^i^	0.97 (2)	2.39 (1)	3.161 (3)	137 (1)
O4—H4⋯O11^ii^	0.97 (2)	2.39 (1)	3.161 (3)	137 (1)

Symmetry codes: (i) 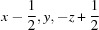; (ii) 
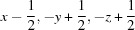
.

**Fig. 1.**
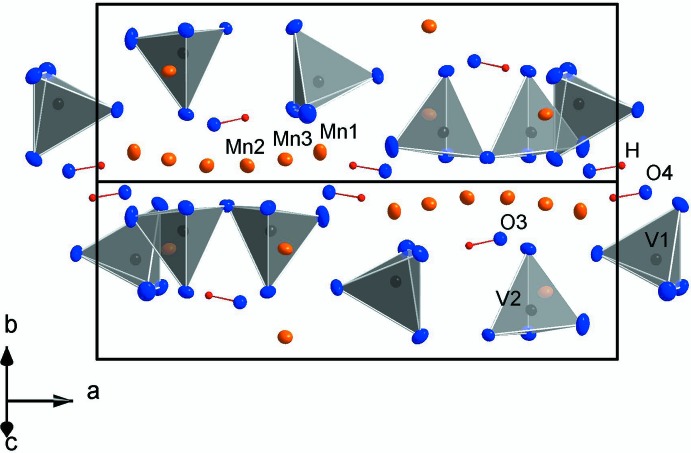
A projection of the crystal structure of Mn2(OH)[VO4] along [011]. Displacement ellipsoids are drawn at the 75% probability level.

**Fig. 2.**
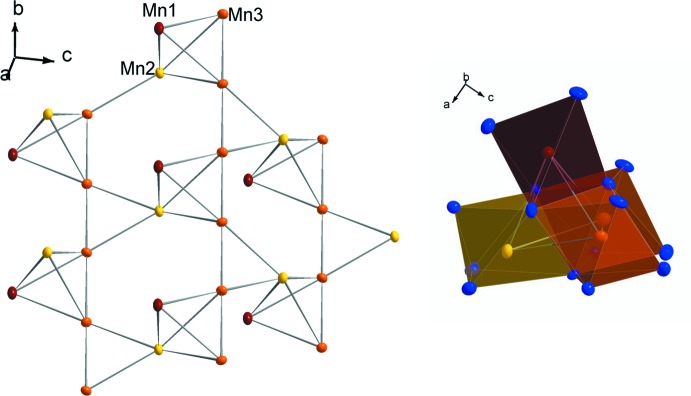
The Mn–Mn connectivity per layer with each connecting line representing a link exclusively via edge-sharing (left). Polyhedral representation for Mn1 (red), Mn2 (yellow) and Mn3 (orange) (right). Displacement ellipsoids are drawn at the 75% probability level.

## supplementary crystallographic information

### S1. Comment

Dimanganese hydroxide vanadate, Mn_2_(OH)[VO_4_], is isotypic with Zn_2_(OH)[VO_4_] (Wang *et al.*, 1998), Zn_1.86_Cd_0.14_(OH)[VO_4_] (Đorđević *et al.*, 2010) and Cu_2_(OH)[VO_4_] (Wu *et al.*, 2003). The crystal structure contains three crystallographically independent Mn^2+^-ions (Fig. 1). Mn3 (8*d*) is located on a general position whereas Mn1 and Mn2 occupy a 4*c* position each, which is of .*m.* site symmetry. Mn1 is found in a distorted trigonal-bipyramidal coordination and connects to Mn2 and two Mn3 only *via* the edges of a single trigonal face. The coordination around Mn2 and Mn3 is octahedral. The former shares four common edges with Mn3 on two *trans*-faces as well as a single edge with Mn1. Mn3 connects to symmetry-related Mn3 positions *via trans*-edges along [010] and through edge-sharing to two Mn2 and only one Mn1. From this connectivity corrugated layers parallel to (100) with terminal [Mn1O_5_] units result (Fig. 2). Per unit cell two of these layers are present which are linked through Mn—O—V—O—Mn bridges into a three-dimensional framework.

Two [VO_4_]^3-^ units are present. Each V-atom and two O per tetrahedron are located on a mirror plane, whereas one O atom per [VO_4_]-unit is found on a general position, respectively. V1 is coordinated by O11 (8*d*), O12 (4*c*), and O13 (4*c*). Mn2 and Mn3 are linked *via* O11 whereas O13 connects to two Mn3. O12 connects solely to Mn1. However, the second coordination sphere around V2 is dissimilar with respect to O21 (8*d*) connecting to Mn1–3 and O23 (4*c*) linked to Mn2 and two Mn3. Again O22 (4*c*) connects only to Mn1. The two hydroxide groups are of .*m.* point group symmetry with one straight and one bifurcated O^D^···O^A^ contacts, respectively (Table 1).

Based on the evaluation of the Mn—O—Mn connectivities, the difference in the second coordination sphere between the [V1O_4_] and [V2O_4_] units, and the dissimilarities found for the two independent hydroxide groups, the more informative structure-related formula should be presented as Mn_4_(OH)_2_[VO_4_]_2_ with *Z* = 4 per unit cell.

It is noteworthy, that for Cu_2_(OH)[VO_4_] two polymorphs (*Pnma* and *P*2_1_2_1_2_1_) were reported by Wu *et al.* (2003) and Zhang *et al.* (2014), respectively. We have also repeatedly obtained the acentric modification (hydrothermal conditions at 493 K, pH 9–10, NH_4_VO_3_ and Cu-acetate or -chloride). Interestingly, Wang *et al.* (1998) include in a side note that Ni_2_(OH)[VO_4_] crystallizes in the *P*2_1_2_1_2_1_ space group as well. Thus, polymorphismn might be related to a size effect or driven by anisotropic magnetic correlations. The latter conjecture is based on the non-magnetic Zn-compound and the "isotropic" *S*=5/2 spin-system represented by Mn^2+^. For these compounds only the centrosymmetric modification are known up to now.

### S2. Experimental

The title compound was obtained as red needle-shaped crystals from reacting H_2_V_3_O_8_ (hydrothermal synthesis, 0.5 mmol V_2_O_5_ and 0.5 mmol H_2_C_2_O_4_ at 453 K) with 1.75 mmol Mn(acetate)_2_^.^4H_2_O and 5 mmol LiCl in 20 ml of water. Concentrated NH_3_-solution was added to adjust the pH to 9.1. The reaction was carried out in a 26 ml Teflon-lined stainless steel autoclave at 493 K for 3 days with subsequent cooling (6 K/h) to room temperature. The final product was washed with distilled water and ethanol alcohol. Source of materials: Mn(acetate)_2_^.^4H_2_O, Sigma; anhydrous H_2_C_2_O_4_, GFS Chemicals; anhydrous LiCl 98+%, Alfa Aesar; V_2_O_5_, Alfa Aesar; NH_4_OH 14.8*M*, EMD.

### S3. Refinement

Hydrogen atom positions were found from difference Fourier maps and were refined by restricting the O—H distance (*DFIX* 1.0 0.02 O3 H3 O4 H4) and using the ride-on option for the isotropic displacements with *U*_iso_(H) = 1.5 × *U*_eq_(O).

### Crystal data

**Table:**

Mn_2_(OH)[VO_4_]	*F*(000) = 912
*M**_r_* = 241.83	*D*_x_ = 3.840 Mg m^−^^3^
Orthorhombic, *P**n**m**a*	Mo *K*α radiation, λ = 0.71075 Å
Hall symbol: -P 2ac 2n	Cell parameters from 18226 reflections
*a* = 14.9112 (10) Å	θ = 3.3–33.1°
*b* = 6.1225 (3) Å	µ = 8.04 mm^−^^1^
*c* = 9.1635 (4) Å	*T* = 293 K
*V* = 836.57 (8) Å^3^	Needle, red
*Z* = 8	0.14 × 0.02 × 0.02 mm

### Data collection

**Table:**

Rigaku R-AXIS conversion diffractometer	1715 independent reflections
Radiation source: fine-focus sealed tube	1637 reflections with *I* > 2σ(*I*)
Graphite monochromator	*R*_int_ = 0.046
Detector resolution: 10.0000 pixels mm^-1^	θ_max_ = 33.1°, θ_min_ = 3.5°
profile data from ω–scans	*h* = −22→22
Absorption correction: multi-scan (*CrystalClear*; Rigaku, 2011)	*k* = −9→9
*T*_min_ = 0.777, *T*_max_ = 1.000	*l* = −13→14
11657 measured reflections	

### Refinement

**Table:**

Refinement on *F*^2^	Primary atom site location: structure-invariant direct methods
Least-squares matrix: full	Secondary atom site location: difference Fourier map
*R*[*F*^2^ > 2σ(*F*^2^)] = 0.045	Hydrogen site location: difference Fourier map
*wR*(*F*^2^) = 0.062	H atoms treated by a mixture of independent and constrained refinement
*S* = 1.36	*w* = 1/[σ^2^(*F*_o_^2^) + (0.0121*P*)^2^ + 1.9324*P*] where *P* = (*F*_o_^2^ + 2*F*_c_^2^)/3
1715 reflections	(Δ/σ)_max_ < 0.001
92 parameters	Δρ_max_ = 0.74 e Å^−^^3^
2 restraints	Δρ_min_ = −0.84 e Å^−^^3^

### Special details

**Table:**

Geometry. All e.s.d.'s (except the e.s.d. in the dihedral angle between two l.s. planes) are estimated using the full covariance matrix. The cell e.s.d.'s are taken into account individually in the estimation of e.s.d.'s in distances, angles and torsion angles; correlations between e.s.d.'s in cell parameters are only used when they are defined by crystal symmetry. An approximate (isotropic) treatment of cell e.s.d.'s is used for estimating e.s.d.'s involving l.s. planes.
Refinement. Refinement of *F*^2^ against ALL reflections. The weighted *R*-factor *wR* and goodness of fit *S* are based on *F*^2^, conventional *R*-factors *R* are based on *F*, with *F* set to zero for negative *F*^2^. The threshold expression of *F*^2^ > σ(*F*^2^) is used only for calculating *R*-factors(gt) *etc*. and is not relevant to the choice of reflections for refinement. *R*-factors based on *F*^2^ are statistically about twice as large as those based on *F*, and *R*-factors based on ALL data will be even larger.

### Fractional atomic coordinates and isotropic or equivalent isotropic displacement parameters (Å^2^)

**Table:**

	*x*	*y*	*z*	*U*_iso_*/*U*_eq_	
Mn1	0.07126 (4)	0.2500	0.08355 (7)	0.01159 (13)	
Mn2	0.28875 (4)	0.2500	0.15984 (6)	0.01046 (12)	
Mn3	0.36181 (3)	0.00226 (7)	−0.12336 (5)	0.01003 (9)	
V1	0.42573 (4)	0.7500	0.18749 (7)	0.00722 (12)	
V2	0.16571 (4)	0.7500	0.02724 (7)	0.00691 (12)	
O11	0.37896 (13)	0.5171 (3)	0.1122 (2)	0.0134 (4)	
O12	0.0956 (2)	0.2500	−0.1322 (3)	0.0170 (6)	
O13	0.46095 (19)	0.2500	−0.1544 (3)	0.0137 (6)	
O21	0.17131 (13)	−0.0131 (3)	0.1339 (2)	0.0111 (4)	
O22	−0.06661 (19)	0.2500	0.0537 (4)	0.0189 (6)	
O23	0.25215 (17)	0.2500	0.3867 (3)	0.0082 (5)	
O3	0.27510 (18)	0.2500	−0.0716 (3)	0.0100 (5)	
H3	0.2151 (17)	0.2500	−0.110 (5)	0.015*	
O4	0.05393 (18)	0.2500	0.3099 (3)	0.0106 (5)	
H4	−0.0080 (16)	0.2500	0.341 (5)	0.016*	

### Atomic displacement parameters (Å^2^)

**Table:**

	*U*^11^	*U*^22^	*U*^33^	*U*^12^	*U*^13^	*U*^23^
Mn1	0.0092 (2)	0.0174 (3)	0.0081 (3)	0.000	−0.0003 (2)	0.000
Mn2	0.0112 (3)	0.0121 (3)	0.0080 (2)	0.000	0.0020 (2)	0.000
Mn3	0.01168 (18)	0.00767 (17)	0.01075 (18)	0.00054 (14)	0.00104 (15)	−0.00112 (15)
V1	0.0066 (2)	0.0081 (3)	0.0070 (3)	0.000	0.0004 (2)	0.000
V2	0.0068 (3)	0.0071 (3)	0.0068 (3)	0.000	0.0012 (2)	0.000
O11	0.0142 (9)	0.0143 (9)	0.0116 (9)	−0.0049 (8)	−0.0008 (8)	−0.0010 (8)
O12	0.0171 (14)	0.0240 (16)	0.0100 (13)	0.000	−0.0001 (12)	0.000
O13	0.0081 (12)	0.0102 (12)	0.0228 (15)	0.000	0.0031 (11)	0.000
O21	0.0132 (8)	0.0105 (9)	0.0095 (9)	0.0002 (7)	0.0027 (7)	−0.0017 (7)
O22	0.0096 (12)	0.0270 (17)	0.0201 (15)	0.000	−0.0036 (12)	0.000
O23	0.0087 (11)	0.0092 (12)	0.0067 (11)	0.000	−0.0020 (10)	0.000
O3	0.0095 (11)	0.0093 (12)	0.0113 (12)	0.000	−0.0005 (11)	0.000
O4	0.0087 (12)	0.0112 (13)	0.0118 (13)	0.000	0.0004 (11)	0.000

### Geometric parameters (Å, º)

**Table:**

Mn1—O12	2.010 (3)	Mn3—O13	2.137 (2)
Mn1—O22	2.074 (3)	Mn3—O11^i^	2.177 (2)
Mn1—O4	2.091 (3)	Mn3—O21^ii^	2.2793 (18)
Mn1—O21^i^	2.243 (2)	Mn3—O23^ii^	2.2981 (19)
Mn1—O21	2.243 (2)	V1—O12^iii^	1.683 (3)
Mn2—O3	2.131 (3)	V1—O13^iv^	1.717 (3)
Mn2—O23	2.149 (3)	V1—O11	1.731 (2)
Mn2—O11	2.162 (2)	V1—O11^v^	1.731 (2)
Mn2—O11^i^	2.162 (2)	V2—O22^vi^	1.654 (3)
Mn2—O21	2.391 (2)	V2—O21^vii^	1.7513 (19)
Mn2—O21^i^	2.391 (2)	V2—O21^i^	1.7513 (19)
Mn3—O3	2.0487 (18)	V2—O23^viii^	1.777 (3)
Mn3—O4^ii^	2.0827 (19)		
			
O12—Mn1—O22	92.83 (13)	O21^ii^—Mn3—O23^ii^	84.16 (8)
O12—Mn1—O4	176.71 (12)	O12^iii^—V1—O13^iv^	111.06 (15)
O22—Mn1—O4	90.46 (12)	O12^iii^—V1—O11	108.39 (9)
O12—Mn1—O21^i^	94.73 (8)	O13^iv^—V1—O11	109.04 (9)
O22—Mn1—O21^i^	133.34 (5)	O12^iii^—V1—O11^v^	108.39 (9)
O4—Mn1—O21^i^	82.99 (7)	O13^iv^—V1—O11^v^	109.04 (9)
O12—Mn1—O21	94.73 (8)	O11—V1—O11^v^	110.93 (14)
O22—Mn1—O21	133.34 (5)	O22^vi^—V2—O21^vii^	107.05 (9)
O4—Mn1—O21	82.99 (7)	O22^vi^—V2—O21^i^	107.05 (9)
O21^i^—Mn1—O21	91.77 (10)	O21^vii^—V2—O21^i^	111.86 (13)
O3—Mn2—O23	159.81 (11)	O22^vi^—V2—O23^viii^	106.89 (15)
O3—Mn2—O11	81.85 (7)	O21^vii^—V2—O23^viii^	111.82 (8)
O23—Mn2—O11	110.69 (7)	O21^i^—V2—O23^viii^	111.82 (8)
O3—Mn2—O11^i^	81.85 (7)	V1—O11—Mn2	142.49 (11)
O23—Mn2—O11^i^	110.69 (7)	V1—O11—Mn3^i^	119.19 (10)
O11—Mn2—O11^i^	98.30 (11)	Mn2—O11—Mn3^i^	94.93 (8)
O3—Mn2—O21	80.28 (7)	V1^viii^—O12—Mn1	158.72 (19)
O23—Mn2—O21	84.84 (7)	V1^iv^—O13—Mn3	134.78 (5)
O11—Mn2—O21	160.92 (7)	V1^iv^—O13—Mn3^i^	134.78 (5)
O11^i^—Mn2—O21	85.76 (7)	Mn3—O13—Mn3^i^	90.43 (11)
O3—Mn2—O21^i^	80.28 (7)	V2^ix^—O21—Mn1	116.62 (10)
O23—Mn2—O21^i^	84.84 (7)	V2^ix^—O21—Mn3^x^	123.94 (10)
O11—Mn2—O21^i^	85.76 (7)	Mn1—O21—Mn3^x^	92.06 (7)
O11^i^—Mn2—O21^i^	160.92 (7)	V2^ix^—O21—Mn2	130.42 (10)
O21—Mn2—O21^i^	84.69 (10)	Mn1—O21—Mn2	91.37 (7)
O3—Mn3—O4^ii^	176.11 (11)	Mn3^x^—O21—Mn2	92.42 (7)
O3—Mn3—O13	86.67 (8)	V2^vi^—O22—Mn1	160.9 (2)
O4^ii^—Mn3—O13	94.01 (8)	V2^iii^—O23—Mn2	121.73 (14)
O3—Mn3—O11^i^	83.40 (9)	V2^iii^—O23—Mn3^x^	122.58 (9)
O4^ii^—Mn3—O11^i^	100.35 (9)	Mn2—O23—Mn3^x^	98.57 (9)
O13—Mn3—O11^i^	95.13 (10)	V2^iii^—O23—Mn3^xi^	122.58 (9)
O3—Mn3—O21^ii^	93.88 (9)	Mn2—O23—Mn3^xi^	98.57 (9)
O4^ii^—Mn3—O21^ii^	82.29 (9)	Mn3^x^—O23—Mn3^xi^	84.45 (9)
O13—Mn3—O21^ii^	89.97 (10)	Mn3^i^—O3—Mn3	95.53 (11)
O11^i^—Mn3—O21^ii^	174.06 (7)	Mn3^i^—O3—Mn2	99.80 (10)
O3—Mn3—O23^ii^	91.24 (7)	Mn3—O3—Mn2	99.80 (10)
O4^ii^—Mn3—O23^ii^	87.68 (7)	Mn3^xi^—O4—Mn3^x^	95.73 (11)
O13—Mn3—O23^ii^	173.63 (10)	Mn3^xi^—O4—Mn1	102.51 (10)
O11^i^—Mn3—O23^ii^	90.60 (9)	Mn3^x^—O4—Mn1	102.51 (10)

Symmetry codes: (i) *x*, −*y*+1/2, *z*; (ii) −*x*+1/2, −*y*, *z*−1/2; (iii) −*x*+1/2, −*y*+1, *z*+1/2; (iv) −*x*+1, −*y*+1, −*z*; (v) *x*, −*y*+3/2, *z*; (vi) −*x*, −*y*+1, −*z*; (vii) *x*, *y*+1, *z*; (viii) −*x*+1/2, −*y*+1, *z*−1/2; (ix) *x*, *y*−1, *z*; (x) −*x*+1/2, −*y*, *z*+1/2; (xi) −*x*+1/2, *y*+1/2, *z*+1/2.

### Hydrogen-bond geometry (Å, º)

**Table:**

*D*—H···*A*	*D*—H	H···*A*	*D*···*A*	*D*—H···*A*
O3—H3···O12	0.96 (2)	1.79 (2)	2.734 (4)	165 (5)
O4—H4···O11^xii^	0.97 (2)	2.39 (1)	3.161 (3)	137 (1)
O4—H4···O11^xiii^	0.97 (2)	2.39 (1)	3.161 (3)	137 (1)

Symmetry codes: (xii) *x*−1/2, *y*, −*z*+1/2; (xiii) *x*−1/2, −*y*+1/2, −*z*+1/2.
